# Trans men’s transition process: gender stereotypes, interventions and experiences

**DOI:** 10.1590/1980-220X-REEUSP-2024-0164en

**Published:** 2025-04-28

**Authors:** Bruno Torelli de Camargo, Flávio Adriano Borges, José Francisco Sampaio Souza, Natália Sevilha Stofel, Diene Monique Carlos, Márcia Niituma Ogata

**Affiliations:** 1 Universidade Federal de São Carlos, Departamento de Enfermagem, São Carlos, SP, Brazil.; 2 Universidade de São Paulo, Escola de Enfermagem de Ribeirão Preto, Ribeirão Preto, SP, Brazil.

**Keywords:** Sexual and Gender Minorities, Transgender Persons, Nursing, Public Health, Institutional Analysis

## Abstract

**Objective:**

To analyze trans men’s perception about the transition process.

**Method:**

A qualitative study, developed with transmasculine people from February to March 2024. A questionnaire and semi-structured interviews were used, which were recorded and transcribed, and analyzed by content analysis. The findings were discussed with five principles of institutional analysis, such as instituted, instituting, institutionalization, implication and over-implication.

**Results:**

Eighteen people participated in the interviews, and data analysis enabled the construction of four classes: i) Gender stereotypes in the social context; ii) Physical interventions and their contexts; iii) Personal and social experiences and relationships with healthcare professionals; iv) Suffering related to passability.

**Conclusion:**

There is no dissociation between social and personal influences in the transition process. They hope that gender-affirming procedures will reduce dysphoria and promote harmony between self-perception and hetero-perception. The acquired passability can strengthen the ability to deal with violence. Furthermore, a broad and solid support network can favor the search for these transitions, facilitating dialogue on gender issues.

## INTRODUCTION

The first mention of transsexuality in literature was made by German physician Magnus Hirschfeld in 1910, when he published a study on 100 cases of transvestites entitled “*Die Travestiten*”. In this work, he introduced the term “psychic transsexualism”. This was the first attempt to understand transsexuality from a medical and sociological perspective, transforming it from a marginalized individual experience into a public health problem subject to medical intervention, including sex reassignment surgeries and hormone treatments^(1)^.

According to the perspective of biological essentialism, which prevailed and still prevails in much of society, there is a correspondence between sex and gender, in which gender is seen as a consequence of sex (cisgenderism). Furthermore, it is assumed that sex determines sexual orientation, with heterosexuality considered the default due to human reproduction. This logic, known as cisheteronormativity, seeks to control bodies under the justification of it being something natural and biological, ignoring cultural and social influences^(2)^.

As for transsexuality, also known and adopted in this production as the expression “trans”, there is no fixed standard of identity. It is defined by a person’s experience and gender identification, which may be different from that assigned at birth^(3)^.

For a long time, transsexuality was considered a pathology and included in the International Classification of Diseases (ICD). Only in 2018, with the ICD-11, transsexuality was removed from the list of mental disorders, being classified as gender incongruence. This change standardized trans gender identity globally, although it was maintained in the ICD to guarantee access to medical treatments (ICD-10: F-64, ICD-11: HA60), seeking to achieve the specific objective VI of the Brazilian National Policy for Comprehensive Health of Lesbians, Gays, Bisexuals, Transvestites and Transsexuals of guaranteeing access to the transsexualization process in the Brazilian Health System (In Portuguese, *Sistema* Único *de Saúde* - SUS) network^(4)^.

Transmasculine identity should not be reduced to the search for medical or surgical transitions, but rather seen as an issue that challenges the boundaries between sex and gender, seeking a broader and more fluid understanding of gender identity^(5)^, i.e., an identity that goes beyond the perspective of a fixed and immutable nature and that considers the existence of a hybridism capable of crossing the very domains of nature and culture artificially established^(6)^.

Belgian estimates indicate that there is a ratio of 1:12,900 (0.07) in scientific literature on trans women and 1:33,800 (0.02) on trans men, but there are no formal studies that determine this estimate concretely^(7)^. In Brazil, scientific productions dealing with healthcare for transgender men are still discreet and recent^(8,9,10,11)^, with the production of information outside the scientific circle being evident through bulletins, reports, guides and other autonomous productions, which show that this population is also placed on the margins of scientific knowledge production.

It is known that transgender people tend to avoid seeking healthcare even when they are sick^(10,12,13)^ or abandon the proposed treatment due to fear of discrimination by healthcare professionals^(14)^. Moreover, access to healthcare services by the transgender population is permeated by constraints and prejudices, highlighting exclusion, helplessness, omission and indifference as the main feelings expressed by these people^(10,14,15)^.

Not only because of this, but also the vast majority of trans men prefer to hide the fact that they are transgender and live as a cis man, enjoying their “passability”, i.e., the ability to be identified through gender expression as a cis man and, thus, obtain respect, protection and basic rights^(5)^, in addition to self-administering testosterone-based steroids without proper medical or health supervision^(16)^.

Given this context, the question is: how do trans men perceive the medical and social transition process? This study is crucial to broaden understanding on the subject, bringing to light the perspective of people who experience the issues of the gender transition process on a daily basis. Therefore, this study aimed to analyze trans men’s perception about the transition process.

## MÉTODO

### Study Design

This is a descriptive and exploratory study with a qualitative approach, which followed the recommendations indicated by the COnsolidated criteria for REporting Qualitative research in its development and sought meanings that articulated theory, practice and further research, in addition to providing an observation of multiple meanings, motives, aspirations, beliefs, values and attitudes, providing the possibility for understanding a deeper space of relationships, processes and phenomena^(17)^.

### Location, Population and Selection Criteria and Sample Definition

The research was conducted in Brazil, with trans men and/or non-binary trans people aligned with the masculine gender. The inclusion criteria for participants in the study were being a trans man and/or non-binary trans person aligned with the masculine gender, residing in Brazil and having or not undergone transition procedures. And the exclusion criteria were not filling out the research form in full, leaving it incomplete and the lack of response to the interview scheduling email after the fifth attempt.

It occurred after its dissemination through the researchers’ social networks (Facebook^®^, Instagram^®^ and WhatsApp^®^), followed by the snowball technique, which uses reference chains for its execution, i.e., after a participant completed the survey, they recommended other participants to the research, disseminating the form through the same link made available to them^(18)^.

This technique can be used in studies with groups that are difficult to access or when the investigation concerns private matters. This research meets both criteria for using this technique, which takes advantage of subjects’ contact network to provide the researcher with a larger set of potential contacts^(18)^. In addition to this, it is understood that snowballing is a technique capable of ensuring that individuals are not exposed, preserving participants’ decision-making capacity, reinforcing respect for diversity and the participation of vulnerable and discriminated groups, such as trans men.

The sample was finalized using the data saturation criterion, i.e., until there was no new topics and information – from the interviews – to the analysis framework of the object of study. Data saturation was achieved when a combination of data empirical limits was identified, their relationship with the theoretical framework of the research and their articulation with the sensitivity of the researchers who developed it^(19)^. In this way, data saturation was achieved in compliance with methodological and scientific rigor, which permeates continuous data analysis (from the beginning of the data collection process), and this preliminary analysis seeks exactly the moment in which little substantial novelty will appear, with the aim of adding to the study objective, answering its guiding question^(19)^.

### Data Collection

Data collection was carried out from February to March 2024, using a self-administered questionnaire, in electronic format, containing sociodemographic data, questions specific to the object of study, published on social media, and semi-structured interviews conducted remotely (online).

Once the form was completed in full, we contacted participants to schedule a semi-structured interview. The recruitment order for the interview was based on the regions of the country and in the chronological order of completion of the form. In other words, recruitment began with the first participant from the Southeast region, who completed the form, followed by the participant from the Northeast region, and so on, until data saturation was reached.

The questions used in the interviews were previously presented to the research group (which is composed of cis, trans and highly ethnically diverse people) for their improvement so that they could be used in the present research. They were: in your opinion, what leads transmasculine people to undergo medical and surgical procedures? If you want to undergo a gender-affirming procedure, what do you expect from it/them? If not, why not? How is the treatment of healthcare professionals usually with you? It is worth mentioning that these were key questions, which unfolded into other questions in the direction of the object of study.

### Data Analysis and Processing

The interviews lasted an average of 30 minutes, and were recorded and transcribed in full, after which content analysis was carried out^(19)^.

Content analysis enables the construction of categories through semantic groupings of words in sentences. This requires sensitivity and flexibility by the coder, with the aim of capturing the thematic cores capable of composing the meaning of the desired communication^(20)^. It consists of the following stages: 1) pre-analysis, which will include text *corpus* composition, text skimming and definition of provisional hypotheses about the content read; 2) material exploration, in which the data will be coded from the recording units; and 3) treatment of results and interpretation, which consists of the classification of elements based on their similarities and by differentiation, with subsequent grouping, given the common characteristics presented by them^(20)^.

It is worth mentioning that, for stage 2 and part of stage 3, the *Interface de R pour les Analyses Multidimensionnelles de Textes et de Questionnaires* (IRAMUTEQ^®^) was used, using the Reinert Method to construct the Descending Hierarchical Classification (DHC). The software performs an analysis based on grouping words with semantic similarity present in the text *corpus*. This *corpus* is divided into text segments (TS), which consist of small textual fragments that preserve a semantic relationship between them^(21)^. The method organizes lexical forms into classes, assigning relative importance to each of them. The occurrences of each of the classes in DHC were considered based on statistically significant values (p < 0.05).

The findings were compared with some principles of institutional analysis, such as institution and its three moments – instituted, instituting and institutionalization -, implication and over-implication. The institution corresponds to the norms and rules created and established socially, with the instituted being that which is clear and identifiable of this institution; the instituting is that which displaces, moves and provokes the instituted; and institutionalization corresponds to the dialectical process between the instituted and the instituting^(22)^. Implication consists of the relationship that people establish with institutions, which can occur in different ways and perspectives, and what makes the process of analyzing this implication difficult corresponds to over-implication^(22)^.

Still in dialogue with institutional analysis, we assumed that the researcher is never neutral in choosing their object of study, and is always implicated in it, whether from an ideological, libidinal and/or organizational perspective. Thus, the authors here assume their implications with the object of this study, as they are healthcare professionals and researchers in the field of sexual and gender minorities within different perspectives (healthcare, prejudice, violence, etc.) and also because most of the authors are from the LGBT+ community, i.e., lesbians, gays, bisexuals, transgenders and other gender identities or sexual orientations that differ from the hetero-cis-normative standard.

### Ethical Aspects

The research was approved by the *Universidade Federal de São Carlos* Research Ethics Committee, under Opinion 5.985.157 and Certificate of Presentation for Ethical Consideration 63925222.2.0000.5504. It is worth noting that, before completing the questionnaire, participants had access to the Informed Consent Form, expressing their agreement to take part in the research. In order to guarantee participant anonymity in the research, the statements made by participants were identified by the expression “Trans”, followed by a corresponding number.

## RESULTS

The self-administered questionnaire was completed by 88 trans men and/or non-binary trans people aligned with the masculine gender. Of these, three were excluded because they did not meet the study inclusion criteria, and 67 were not recruited for the interview due to data saturation, which occurred in the 18^th^ interview. Therefore, 18 trans men and/or non-binary trans people aligned with the masculine gender, who were before, after or during medical/social transition or who chose not to undergo any of the transitions, participated in the semi-structured interview stage. It is worth mentioning that no participant, when recruited for the interview, refused and/or was excluded from the research (Table 1).

**Table 1:** Sociodemographic characterization of trans men and/or non-binary trans people aligned with the masculine gender who participated in the study – São Carlos, SP, Brazil, 2024.

Sexual orientation	City/state	Income (minimum wage)	Color/ethnicity	Level of education	Age (years)
Pansexual	Rio de Janeiro/RJ	1/2 to 1	Indigenous	Complete higher education	21 to 25
Pansexual	Campinas/SP	1 to 2	Brown	Incomplete higher education	21 to 25
Pansexual	São Paulo/SP	1 to 2	White	Complete higher education	21 to 25
Bisexual	Betim/MG	1/2 to 1	Black	Incomplete higher education	21 to 25
Pansexual	Feira de Santana/BA	2 to 3	White	Complete higher education	21 to 25
Bisexual	Fortaleza/CE	1/2 to 1	Yellow	Incomplete higher education	21 to 25
Asexual	Salvador/BA	No income	Brown	complete high school	16 to 20
Bisexual	Sobral/CE	2 to 3	White	Complete high school	16 to 20
Bisexual	Curitiba/PR	1 to 2	White	Complete graduate degree	26 to 30
Asexual	Passo Fundo/RS	No income	White	Incomplete higher education	21 to 25
Gay	Bocaina do Sul/SC	1/2 to 1	White	Incomplete high school	16 to 20
Pansexual	Florianópolis/SC	No income	White	Incomplete higher education	21 to 25
Gay	Manaus/AM	1 to 2	White	Incomplete higher education	21 to 25
Gay	Belém/PA	1 to 2	White	Complete higher education	16 to 20
Pansexual	Boa Vista/RR	More than 3	White	Incomplete higher education	16 to 20
Heterosexual	Cuiabá/MT	1/2 to 1	Black	Incomplete higher education	21 to 25
Heterosexual	Rondonópolis/MT	2 to 3	White	Complete higher education	16 to 20
Bisexual	Três Lagoas/MS	More than 3	White	Incomplete higher education	16 to 20

Source: research data.

During the text *corpus* parameterization, 18 texts were analyzed, corresponding to the 18 interviews conducted with trans men, and were divided into 854 TSs. Of these, 691 were classified with 80.91%, indicating that the interviewees maintained focus on the main subject and did not present significant vocabulary variations. Furthermore, 28,478 lexical forms were identified in total, of which only 3,578 were distinct and 1,833 of them appeared only once.

**Figure 1:**
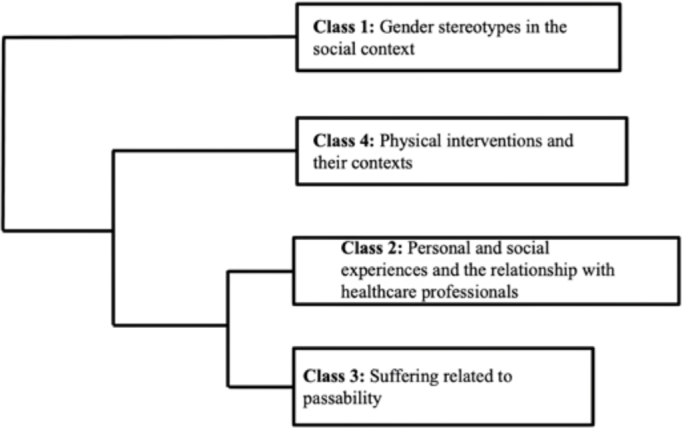
Dendrogram of analysis of the text *corpus* of interviews. São Paulo, Brazil, 2024.

The text *corpus* DHC resulted in the creation of a dendrogram with three partitions. The first partition separated class 1 (“Gender stereotypes in the social context”) from the others. The second partition originated class 4 (“Physical interventions and their contexts”), while the third partition separated classes 2 (“Personal and social experiences and the relationship with healthcare professionals”) and 3 (“Suffering related to passability”) (Figure 1).

Class 1 presented 186 TSs out of the 691 classified by IRAMUTEQ, with a relative importance of 26.9% and 149 active forms. Class 2 included 202 TSs, with a relative importance of 29.2% and 114 active forms. Class 3 had 111 TSs, with a relative importance of 16.1% and 109 active forms. Finally, class 4 included 192 TSs, with a relative importance of 27.8% and 145 active forms.

### Gender Stereotypes in The Social Context

This class brings together the observations made by interviewees regarding gender stereotypes within a social context regarding the performance expected by society of men and women based on their gender identities as well as their characteristics and behaviors. This is demonstrated by the words that constitute the class with a significance value <0.0001, such as “man” (chi2 75.25), “woman” (chi2 66.68), “standard” (chi2 52.99), “society” (chi2 42.53), “pressure” (chi2 26.03) and “appearance” (chi2 16.43).

*Socially seen as a byproduct, man needs to be read as a masculine and virile figure, a brute figure, a figure with an active voice, with characteristics that reinforce this pattern.* (Trans13)

*Society implies that to be a man, you need to have no breasts and a beard. I believe this comes from the gender binary, since from the moment we are born, something and a standard are imposed on us to be followed only by our biological sex.* (Trans5)

*The deconstruction of what we grew up understanding about what it means to be a man or a woman is something that is ongoing, even more so when we talk about people who are non-binary*. (Trans19)

*I believe there must be a lot of pressure from society with this, in the sense that, if you identify as a man, you should have a male body.* (Trans12)

### Personal and Social Experiences and The Relationship With Healthcare Professionals

This class is formed by words with significance < 0.0001, such as “talk” (chi2 54.79), “find” (chi2 37.56), “general” (chi2 18.11) and “head” (chi2 18.11).

The TSs that relate to personal experiences demonstrate that undergoing gender-affirming procedures help in recognizing one’s own body, providing a better quality of life and resources to deal with transphobia. In addition, the larger the support network, the more appropriate the decision to transition is, with more time for reflection and productive discussions on the subject.

*The more I can show that I am a man, without having to open my mouth to say it, the better. At the same time, I want to go through procedures to feel good about myself*. (Trans10)

*I think that when we feel good on a personal level, we become somewhat shielded from external things. Nowadays, things affect me much less.* (Trans10)

*I think support is important, because I know I won’t go through this process alone. It’s not just support at home, but support from friends too.* (Trans3)

The TSs that focus on the social sphere state that, even when trans people undergo gender-affirming procedures to reduce the violence they suffer, it ends up being a decision of self-preservation, which ensures greater mental stability. Not only that, but psychological support is also necessary, because transitioning is not a guarantee that there will be no more issues with one’s own body or transphobia.

*I wouldn’t say I’m a very passable person, I suffer a lot with this issue, but I’ve been trying to understand that the problem isn’t me, but other people.* (Trans7)

*I was a little apprehensive before I started, I won’t deny it. The people around me put it in my head that I would regret it, but I had no doubt about what I wanted.* (Trans9)

*I don’t live in a cave, people assuming something about me makes me feel bad. Like, here in my head.* (Trans3)

In the TSs that refer to healthcare professionals, most reports are positive regarding the adoption of the social name. However, all stated that both professionals and healthcare services themselves, whether public or private, have little or no information about the flow of care for a trans person seeking gender-affirming procedures. Moreover, they demonstrate that healthcare professionals have little or no interest in addressing health issues of trans people that do not relate to procedures provided for in the transsexualization process, which maintains both the prejudices that LGBTQIAP+ people are at risk for various health conditions and the barriers to accessing healthcare for this population.

*Case of trans people requesting to use their social name to take the exams. But, overall, my little experience was very calm.* (Trans11)

*I think there was a lack of sensitivity and information about the processes. The medical community is sometimes a bit insensitive in general.* (Trans8)

*I’m on the right track, but it was a struggle to find an endocrinologist who would treat the trans population in my city*. (Trans9)

### Suffering Related to Passability

This class presents the words “health” (chi2 63.14), “hormonal” (chi2 46.44), “transition” (chi2 38.06), “decision” (chi2 36.95), “safe” (chi2 25.44), “fear” (chi2 23.01) and “aesthetics” (chi2 21.02), with significance < 0.0001. It has STs that talk about how having passability makes trans people receive more respectful treatment from society and also allows them to live more moments free from fear.

*Before I started hormone treatment, I would force my voice to become deeper so much that there were days when I could barely speak because my throat was so sore. I didn’t call my friends normally, I avoided audio as much as possible, and I only talked when absolutely necessary.* (Trans9)

*I don’t regret having made that decision, because interactions with society affected me so much that I was falling into a very strong depression; I no longer had the will to live.* (Trans7)

*I’ve always been and still am very afraid of walking down the street at night, but that has lessened since I’ve become somewhat passable, because now the most that will happen to me is someone stealing my cell phone, not violating me in any other way*. (Trans10)

### Physical Interventions and Their Contexts

The words with significance <0.0001 in this class are “surgery” (chi2 32.65), “expectation” (chi2 88.24), “Binder” (chi2 25.49), “mastectomy” (chi2 19.03), “hormonalization” (chi 75.0) and “pain” (chi2 90.91). This class addresses the scenarios of medical and surgical interventions in terms of their accessibility, the impact they have on trans people’s lives and the expectations associated with transition.

*I wish mastectomies and uterus removal were more accessible surgeries, because they are very expensive. Not to mention the social aspect, that these surgeries are considered plastic surgeries. For me, these surgeries are suicide prevention*. (Trans17)

*I was very happy to have the surgery, but neither the surgery nor the start of hormone therapy was things that made me go, “Oh my God, that’s amazing! I did it!” because I personally feel like I’m just adjusting to my place.* (Trans9)

*Getting dressed for college without a Binder is much worse than any back pain. Dysphoria and social issues definitely play a big role in all of this.* (Trans16)

## DISCUSSION

Gender stereotypes are simplified representations of characteristics and roles attributed to men and women based on their gender identities, established in the capitalist mode of production. Since then, society has demanded the performance of standards, such as female submission and male superposition, and applied specific mechanisms of oppression to those who differ from heterocisnormativity, such as the process of compulsory social marginalization of trans people^(23,24)^. Thus, stereotypes maintain the reproduction of the current mode of production and sociability, influencing various aspects of life with the social rules of femininity and masculinity, which shape behaviors, relationships, social expectations and even professional choices articulated with patriarchy, sexism, racism and other structural prejudices.

This fact is in line with the process of institutionalization of sexuality in the identity, gender expression and sexual orientation dimensions, which maintain socially instituted “rules” and “norms”, in which any body that differs from the heterocisnormative and binary standard acts as an instituting force, provoking and moving this institution. However, this movement produces suffering, pain, lack of healthcare and death.

There are still many barriers to accessing healthcare services for the transmasculine population, with fear of suffering transphobia being the most common factor cited by those interviewed. This is followed by misinformation and lack of preparation of professionals regarding the flow of care for a trans person seeking a gender-affirming procedure, whether in public or private healthcare services. This fact supports research conducted with 116 trans people in the United States, which points to the need to enact health policies capable of deconstructing prejudices in healthcare services to ensure comprehensive and humanized care for this population^(25)^.

However, research conducted with 110 Brazilian nurses revealed the social representation of prejudice surrounding the word “transvestite”^(26)^, which highlights the existence of many healthcare professionals who deny adequate care and referral to trans people due to discrimination, while claiming reasons of belief and/or personal values and also lack of qualifications that, consequently, intensify suffering, social distancing and barriers to healthcare services^(26)^. In addition, there is no concern in making environments welcoming to the transmasculine population, demonstrated by the lack of connection between this population and health teams, which directly affects the effectiveness of health actions^(25,26)^.

This fact is in line with the notion of implication regarding the institution of sexuality, i.e., the relationship that healthcare professionals establish with this institution in their daily work. Sometimes, an implication permeated by dogmas and beliefs may be capable of generating interferences that hinder the process of implication analysis, causing these professionals to maintain a certain resistance and, consequently, become over-implicated in relation to sexuality. In other words, they will be “impermeable” to any instituting force that is capable of provoking their established way of thinking and doing in health with regard to human sexuality.

Another topic that came up in the interviews was raising awareness in the medical community about both general and specific demands related to the transgender process. Although trans people have the right to quality services, effective public policies, and professionals committed to comprehensive care, who listen to them with qualified skills and understand their demands and needs, they still face disservice, especially due to structural problems in the health system and a lack of debates in health education about sexual and gender diversity^(25,27)^.

It is an issue that dialogues with the activism of lesbian and bisexual women, due to the invisibility and abjection of these bodies, precariousness and vulnerability in achieving recognition from healthcare professionals^(28)^. Therefore, many medical/surgical procedures that should end dysphoria and promote passability end up exposing transition and transsexuality, either because they were done clandestinely and with few resources and/or because they were performed by doctors who did not care about bringing harmony to trans bodies and only want to profit from the desperation for the procedure.

This last point says a lot about the medical over-implication of what is established about sexuality, reinforcing compulsory cisgenderism in professional practice, i.e., without there being a reflective and permanent process that reveals the fluidity and transience of human sexuality, and the instituting forces produced daily through the different ways of being and existing in the world.

Body modification practices aim to “conform” a trans person’s body, in order to produce personal satisfaction and achieve the bodily materiality of what they consider to be their ideal body^(27)^. This would be a way of adapting to the established rules regarding binary gender expressions that are compulsorily considered “correct”. Therefore, it is essential that medical-surgical procedures provided for in the SUS Reassignment Process carefully consider the desires and expectations of a trans person, carefully assessing the available resources and, at the same time, recognizing the autonomy, leading role and legitimacy of a person in relation to the procedure by a healthcare professional. It is crucial to maintain a vision that is sensitive to the biopolitics that permeates these bodies, because they will undergo social and symbolic inscriptions^(27,29)^. In fact, they will suffer not only from cisgender society, but also within the trans community itself, where there is a practice of comparing surgeries that encourages competition and judgment, as opposed to celebration and support.

The SUS still faces many challenges in providing adequate care to the trans population. In practice, health teams’ capacity to resolve trans people’s demands is very low, because there is no support network, no financial resources allocated to this issue, no technical preparation of teams that provide care, and no preparation of organizations themselves with regard to adopting daily care flows and protocols^(30)^. Furthermore, the transsexualization process was not designed to be the axis of public policy, and the set of regulations, principles and guidelines are not intended to implement one^(30)^. It consists of the maxim that, for there to be a process of institutionalization in institutions, such as sexuality, people must be involved in this process, since it is a construction that is effectively established and built socially.

It is worth noting that private healthcare services suffer from the same problems. However, in this case, there is also the financial factor and the high cost, which make access unfeasible for the vast majority. As a result, there is considerable clandestine activity related to the sale of hormones and the performance of surgical procedures, in the same way that other healthcare technologies have lost their direct exclusivity with medicine and have begun to be captured by the financial market, often by non-healthcare professional sellers, allowing many people to go through transition processes without assistance^(30)^.

This study brings to light issues related to the transition processes experienced by trans men, which involve seeking medical procedures and care in healthcare services and daily social and individual coping. Given the social ills in which these people are placed and the neoliberal and predatory perspective of developing research on them and never with them, they rarely lead or participate in studies on this topic. Furthermore, this production advances in the look at transmasculine people’s health, highlighting the bottleneck that still exists in Brazilian public health for meeting their health needs and which intercepts several directions, such as the need for a close look at the training of healthcare professionals and the Continuing Education in Health of professionals already trained regarding trans people’s health. It presents the following limitations: the fact that recruitment took place through an online form, which allowed it to be completed by anyone who was not necessarily the target audience of the study; the interviews were conducted remotely (online), which made it extremely difficult to ensure the quality of their participation without interruptions due to personal reasons and/or poor internet quality; and the cultural diversity of research participants, which, while enriching the study, brought biases in relation to the differences that exist in the SUS within the national territory.

## CONCLUSION

Regarding trans men’s perception of the medical and social transition process, this study points to the dissociation between social and personal influences in the transition process experienced by transmasculine people. The issue of appearance is one of the major motivators, especially the physical changes caused by hormone therapy and mastectomy. Thus, the expectations surrounding gender-affirming procedures were to achieve both a state in which there is no longer dysphoria with one’s own body and harmony between self-perception and the way in which one is perceived by others, minimizing incongruent gender assumptions and situations of transphobia as much as possible.

That said, this study identified that transmasculine people seek passability acquired through medical and surgical transitions, expanding their mental resources to deal with episodes of violence, especially with regard to self-blame. Moreover, the existence of a broad and solid support network can favor the search for these transitions, offering emotional support and facilitating dialogue on gender issues.
