# Supplementary material for: Trans men’s transition process: gender stereotypes, interventions and experiences
Source: Rev Esc Enferm USP. 2025 Apr 28;59:e20240164. doi: 10.1590/1980-220X-REEUSP-2024-0164en (PMC12039820; doi:10.1590/1980-220X-REEUSP-2024-0164en)
Supplement: Translation

# Translation: Processo de transição de homens trans: estereótipos de gênero, intervenções e experiências

**DOI:** 10.1590/1980-220X-REEUSP-2024-0164pt

**Authors:** Bruno Torelli de Camargo, Flávio Adriano Borges, José Francisco Sampaio Souza, Natália Sevilha Stofel, Diene Monique Carlos, Márcia Niituma Ogata

**Affiliations:** 1 Universidade Federal de São Carlos, Departamento de Enfermagem, São Carlos, SP, Brasil.; 2 Universidade de São Paulo, Escola de Enfermagem de Ribeirão Preto, Ribeirão Preto, SP, Brasil.

**Keywords:** Minorias Sexuais e de Gênero, Pessoas Transgênero, Enfermagem, Saúde Pública, Análise Institucional

## Abstract

**Objetivo:**

Analisar a percepção de homens trans sobre o processo de transição.

**Método:**

Estudo qualitativo, desenvolvido com pessoas transmasculinas de fevereiro a março de 2024. Foram utilizados um questionário e entrevistas semiestruturadas, que foram gravadas e transcritas, sendo analisadas por análise de conteúdo. Os achados foram dialogados com cinco princípios da análise institucional, como instituído, instituinte, institucionalização, implicação e sobreimplicação.

**Resultados:**

Participaram das entrevistas 18 pessoas, e a análise dos dados possibilitou a construção de quatro classes: i) Estereótipos de gênero no contexto social; ii) Intervenções físicas e seus contextos; iii) Experiências pessoais, sociais e relação com profissionais de saúde; iv) Sofrimentos relacionados à passabilidade.

**Conclusão:**

Não há uma dissociação entre influências sociais e pessoais no processo de transição. Eles esperam que os procedimentos afirmadores de gênero reduzam a disforia e promovam uma harmonia entre autopercepção e heteropercepção. A passabilidade adquirida pode fortalecer a capacidade de lidar com a violência. Ainda, uma rede de apoio ampla e sólida pode favorecer a busca por essas transições, facilitando o diálogo sobre questões de gênero.

## INTRODUÇÃO

A primeira menção à transexualidade na literatura foi feita pelo médico alemão Magnus Hirschfeld, em 1910, quando publicou estudo sobre 100 casos de travestis intitulado “*Die Travestiten*”. Neste trabalho, ele introduziu o termo “transexualismo psíquico”. Essa foi a primeira tentativa de compreender a transexualidade sob uma perspectiva médica e sociológica, transformando-a de uma experiência individual marginalizada em um problema de saúde pública passível de intervenção médica, incluindo cirurgias de redesignação sexual e tratamentos hormonais^(1)^.

De acordo com a perspectiva do essencialismo biológico, que prevaleceu e ainda prevalece em grande parte da sociedade, existe uma correspondência entre sexo e gênero, em que o gênero é visto como uma consequência do sexo (cisgeneridade). Além disso, assume-se que o sexo determina a orientação sexual, sendo a heterossexualidade considerada como padrão devido à reprodução humana. Essa lógica, conhecida como cisheteronormatividade, busca controlar os corpos sob a justificativa de ser algo natural e biológico, ignorando as influências culturais e sociais^(2)^.

Quanto à transexualidade, também conhecida e adotada nesta produção como a expressão “trans”, não há um padrão fixo de identidade. Ela é definida pela experiência e identificação de gênero da pessoa, que pode ser diferente daquela designada ao nascer^(3)^.

Durante muito tempo, a transexualidade foi considerada uma patologia e incluída na Classificação Internacional de Doenças (CID). Somente em 2018, com a CID-11, a transexualidade foi retirada da lista de transtornos mentais, sendo classificada como incongruência de gênero. Essa mudança padronizou a identidade de gênero trans globalmente, embora tenha sido mantida na CID para garantir acesso a tratamentos médicos (CID-10: F-64, CID-11: HA60), buscando alcançar o objetivo específico VI da Política Nacional de Saúde Integral de Lésbicas, Gays, Bissexuais, Travestis e Transexuais de garantia do acesso ao processo transexualizador na rede do Sistema Único de Saúde (SUS)^(4)^.

A identidade transmasculina não deve ser reduzida à busca por transições médicas ou cirúrgicas, mas sim vista como uma questão que desafia as fronteiras entre sexo e gênero, buscando uma compreensão mais ampla e fluida da identidade de gênero^(5)^, ou seja, uma identidade que ultrapasse a perspectiva de uma natureza fixa e imutável e que considere a existência de um hibridismo capaz de atravessar os próprios domínios da natureza e da cultura artificialmente estabelecidos^(6)^.

Estimativas belgas apontam que há de 1:12.900 (0,07) na literatura científica sobre mulheres trans e de 1:33.800 (0,02) sobre homens trans, mas não há estudos formais que determinem essa estimativa concretamente^(7)^. No Brasil, ainda são discretas e recentes as produções científicas tratando sobre a atenção à saúde de homens transexuais^(8,9,10,11)^, sendo evidente a produção de informações fora do círculo científico por meio de boletins, relatórios, guias e demais produções autônomas, que evidenciam que essa população também é colocada à margem da produção do conhecimento científico.

Sabe-se que pessoas transexuais tendem a evitar a procura por atendimento de saúde mesmo estando doentes^(10,12,13)^ ou abandonando o tratamento proposto em decorrência do medo da discriminação pelos profissionais de saúde^(14)^. Somado a isso, o acesso aos serviços de saúde pela população transexual eú permeado por constrangimentos e preconceitos, ressaltando a exclusão, o desamparo, a omissão e a indiferença como os principais sentimentos expressos por essas pessoas^(10,14,15)^.

Não só por isso, mas também a grande maioria dos homens trans prefere omitir que é transgênero e viver como um homem cis, usufruindo da sua “passabilidade”, ou seja, da capacidade de ser identificado por meio da expressão de gênero como um homem cis e, assim, obter respeito, proteção e direitos básicos^(5)^, além de autoadministrar esteroides à base de testosterona sem o devido acompanhamento médico ou de saúde^(16)^.

Diante desse contexto, questiona-se: como os homens trans percebem o processo de transição médica e social Este estudo é crucial para ampliar a compreensão sobre o assunto, trazendo à tona o olhar de pessoas que vivem cotidianamente as questões do processo de transição de gênero. Portanto, este estudo tem como objetivo analisar a percepção de homens trans sobre o processo de transição.

## MÉTODO

### Desenho do Estudo

Trata-se de estudo descritivo e exploratório de abordagem qualitativa, que seguiu as recomendações apontadas pelo *COnsolidated criteria for REporting Qualitative research* em seu desenvolvimento e procurou sentidos que articulassem teoria, prática e mais pesquisa, além de proporcionar uma observação de múltiplos significados, motivos, aspirações, crenças, valores e atitudes, conferindo possibilidade para a compreensão de um espaço mais profundo de relações, processos e fenômenos^(17)^.

### Local, População e Critérios de Seleção e Definição da Amostra

A pesquisa foi desenvolvida no Brasil, com homens trans e/ou pessoas trans não binárias alinhadas ao gênero masculino. Os critérios de inclusão dos participantes no estudo foram ser homem trans e/ou pessoa trans não binária alinhada ao gênero masculino, residente no Brasil e terem ou não passado por procedimentos de transição. E os critérios de exclusão foram não preencher na íntegra o formulário da pesquisa, deixando-o incompleto e a ausência de resposta ao e-mail de agendamento da entrevista após a quinta tentativa.

Ela ocorreu a partir da sua divulgação por meio das redes sociais dos pesquisadores (*Facebook* ^®^, *Instagram* ^®^ e *WhatsApp* ^®)^, seguida da técnica *snowball* (bola de neve), que utiliza cadeias de referência para a sua execução, ou seja, após o preenchimento da pesquisa por um participante, este indicou outros participantes à pesquisa, divulgando o formulário por meio do mesmo *link* disponibilizado a ele^(18)^.

Essa técnica pode ser utilizada em estudos com grupos de difícil acesso ou quando a investigação diz respeito a assuntos privados. Esta pesquisa atende a ambos critérios para utilização dessa técnica, que aproveita da rede de contatos dos sujeitos para fornecer ao pesquisador um conjunto maior de potenciais contatos^(18)^. Somado a isso, compreende-se que a *snowball* é uma técnica capaz de garantir a não exposição de indivíduos, preservando a capacidade de tomada de decisões de participantes, reforçando o respeito à diversidade e a participação de grupos vulnerabilizados e discriminados, como é o caso dos homens trans.

A finalização da amostra ocorreu pelo critério de saturação dos dados, ou seja, até que houvesse a ausência de novos temas e informações – pelas entrevistas – ao quadro de análise do objeto de estudo. A saturação dos dados foi alcançada a partir do momento em que se identificou uma combinação dos limites empíricos dos dados, da relação dos mesmos com o arcabouço teórico da pesquisa e da articulação com a sensibilidade dos pesquisadores que a desenvolveram^(19)^. Dessa forma, a saturação dos dados foi feita obedecendo o rigor metodológico e científico, que perpassa a análise contínua dos dados (desde o início do processo da coleta dos mesmos), sendo que essa análise preliminar busca exatamente pelo momento em que pouca novidade substancial aparecerá, com o intuito de acrescentar ao objetivo do estudo, respondendo à questão norteadora do mesmo^(19)^.

### Coleta de Dados

A coleta dos dados foi realizada de fevereiro a março de 2024, sendo utilizados questionário autoaplicado, em formato eletrônico, contendo dados sociodemográfico, questões específicas ao objeto de estudo, divulgado em mídias sociais, e entrevistas semiestruturadas realizadas remotamente (*online*).

A partir do preenchimento do formulário na íntegra, entramos em contato com os participantes buscando realizar o agendamento da entrevista semiestruturada. A ordem de recrutamento para a entrevista se deu a partir das regiões do país e obedecendo a ordem cronológica de preenchimento do formulário, ou seja, iniciou-se o recrutamento com o primeiro participante da região Sudeste, que preencheu o formulário, em seguida o da região Nordeste, e assim por diante, até alcançar a saturação dos dados.

As questões utilizadas nas entrevistas foram previamente apresentadas ao grupo de pesquisa (que possui, em sua composição, pessoas cis, trans e grande diversidade étnico-racial), em prol do seu aperfeiçoamento, para que pudessem sem empregadas na presente pesquisa, sendo elas: na sua opinião, o que leva pessoas transmasculinas a realizarem procedimentos médicos cirúrgicos Se você deseja passar por algum procedimento afirmador de gênero, o que você espera dele(s) Se não, por que não Como costuma ser o tratamento de profissionais de saúde com você Vale constar que se trataram de questões-chave, que se desdobraram em outros questionamentos na direção do objeto do estudo.

### Análise e Tratamento dos Dados

As entrevistas tiveram duração média de 30 minutos, e foram gravadas e transcritas na íntegra, procedendo-se com a análise de conteúdo^(19)^.

A análise de conteúdo possibilita a construção de categorias por meio de agrupamentos semânticos das palavras nas frases. Tal fato exige sensibilidade e flexibilidade por parte do codificador, com o intuito de apreender os núcleos temáticos capazes de compor o sentido da comunicação desejada^(20)^. Ela é composta pelas seguintes etapas: 1) pré-análise, que contará com a composição do *corpus* textual, leitura flutuante e definição de hipóteses provisórias sobre o conteúdo lido; 2) exploração do material, em que os dados serão codificados a partir das unidades de registro; e 3) tratamento dos resultados e interpretação, que consiste na classificação dos elementos a partir das suas semelhanças e por diferenciação, com posterior agrupamento, diante das características comuns apresentadas pelos mesmos^(20)^.

Vale ressaltar que, para a etapa 2 e parte da etapa 3, foi utilizado o auxílio do *software Interface de R pour les Analyses Multidimensionnelles de Textes et de Questionnaires* (IRAMUTEQ^®^), utilizando o Método de Reinert para construir a Classificação Hierárquica Descendente (CHD). O *software* realiza uma análise a partir do agrupamento de vocábulos com similaridade semântica presentes no corpus textual. Esse corpus é dividido em segmentos de textos (STs), que consistem em pequenos fragmentos textuais que preservam uma relação semântica entre si^(21)^. O método organiza as formas lexicais em classes, atribuindo importância relativa a cada uma delas. As ocorrências de cada uma das classes na CHD foram consideradas a partir de valores estatisticamente significantes (p < 0,05).

Os achados obtidos foram confrontados com alguns princípios da análise institucional, tais como instituição e seus três momentos – instituído, instituinte e institucionalização -, implicação e sobreimplicação. A instituição corresponde às normas e regras criadas e estabelecidas socialmente, sendo o instituído aquilo que é nítido e identificável dessa instituição; o instituinte é aquilo que desloca, movimenta e provoca o instituído; e a institucionalização corresponde ao processo dialético entre o instituído e o instituinte^(22)^. Já a implicação consiste na relação que as pessoas estabelecem com as instituições, que pode ocorrer de diferentes formas e perspectivas, e aquilo que dificulta o processo de análise dessa implicação corresponde à sobreimplicação^(22)^.

Ainda em diálogo com a análise institucional, partimos do pressuposto de que o pesquisador nunca é neutro na escolha do seu objeto de estudo, estando sempre implicado com o mesmo, seja por uma perspectiva ideológica, libidinal e/ou organizacional. Assim, os(as) autores(as) assumem aqui suas implicações com o objeto deste estudo, por serem profissionais e pesquisadores(as) da área da saúde na área das minorias sexuais e de gênero dentro de diferentes perspectivas (atenção à saúde, preconceito, violência, etc.) e também pela maior parte dos(as) autores(as) serem da comunidade LGBT+, ou seja, lésbicas, gays, bissexuais, transgêneros e demais identidades de gênero ou orientação sexual que difere do padrão hétero-cis-normativo.

### Aspectos Éticos

A pesquisa foi aprovada pelo Comitê de Ética em Pesquisa com Seres Humanos da Universidade Federal de São Carlos, sob Parecer n° 5.985.157 e Certificado de Apresentação para Apreciação Ética n° 63925222.2.0000.5504. Vale apontar que, antes do preenchimento do questionário, o participante teve acesso ao Termo de Consentimento Livre e Esclarecido, manifestando sua concordância em fazer parte da pesquisa. Com o intuito de garantir o anonimato dos participantes na pesquisa, as falas proferidas pelos participantes foram identificadas por meio da expressão “Trans”, seguida de um número correspondente.

## RESULTADOS

Preencheram o questionário autoaplicado 88 homens trans e/ou pessoas trans não binárias alinhadas ao gênero masculino. Desses, três foram excluídos em decorrência de não se enquadrarem nos critérios de inclusão do estudo, e 67 não chegaram a ser recrutados para a entrevista em decorrência da saturação dos dados, que ocorreu na 18^a^ entrevista. Portanto, 18 homens trans e/ou pessoas trans não binárias alinhadas ao gênero masculino, que se encontravam antes, após ou durante transição médica/social ou que optaram por não realizar alguma das transições, participaram da etapa da entrevista semiestruturada. Vale ressaltar que nenhum participante, quando recrutado para a entrevista, negou e/ou foi excluído da pesquisa (Tabela 1).

**Tabela 1:** Caracterização sociodemográfica de homens trans e/ou pessoas trans não binárias alinhadas ao gênero masculino que participaram do estudo – São Carlos, SP, Brasil, 2024.

Orientação sexual	Cidade/estado	Renda (salário mínimo)	Cor/etnia	Grau de escolaridade	Idade (anos)
Pansexual	Rio de Janeiro/RJ	1/2 a 1	Indígena	Ensino superior completo	21 a 25
Pansexual	Campinas/SP	1 a 2	Parda	Ensino superior incompleto	21 a 25
Pansexual	São Paulo/SP	1 a 2	Branca	Ensino superior completo	21 a 25
Bissexual	Betim/MG	1/2 a 1	Preta	Ensino superior incompleto	21 a 25
Pansexual	Feira de Santana/BA	2 a 3	Branca	Ensino superior completo	21 a 25
Bissexual	Fortaleza/CE	1/2 a 1	Amarela	Ensino superior incompleto	21 a 25
Assexual	Salvador/BA	Sem renda	Parda	Ensino médio completo	16 a 20
Bissexual	Sobral/CE	2 a 3	Branca	Ensino médio completo	16 a 20
Bissexual	Curitiba/PR	1 a 2	Branca	Pós-graduação completa	26 a 30
Assexual	Passo Fundo/RS	Sem renda	Branca	Ensino superior incompleto	21 a 25
Gay	Bocaina do Sul/SC	1/2 a 1	Branca	Ensino médio incompleto	16 a 20
Pansexual	Florianópolis/SC	Sem renda	Branca	Ensino superior incompleto	21 a 25
Gay	Manaus/AM	1 a 2	Branca	Ensino superior incompleto	21 a 25
Gay	Belém/PA	1 a 2	Branca	Ensino superior completo	16 a 20
Pansexual	Boa Vista/RR	Mais de 3	Branca	Ensino superior incompleto	16 a 20
Heterossexual	Cuiabá/MT	1/2 a 1	Preta	Ensino superior incompleto	21 a 25
Heterossexual	Rondonópolis/MT	2 a 3	Branca	Ensino superior completo	16 a 20
Bissexual	Três Lagoas/MS	Mais de 3	Branca	Ensino superior incompleto	16 a 20

Fonte: dados da pesquisa.

Durante a parametrização do corpus textual, foram analisados 18 textos, que correspondem às 18 entrevistas realizadas com homens trans, e foram divididos em 854 STs. Desses, 691 foram classificados com 80,91%, indicando que os entrevistados mantiveram o foco no assunto principal e não apresentaram variações vocabulares significativas. Além disso, foram identificadas 28.478 formas lexicais no total, das quais apenas 3.578 eram distintas e 1.833 delas apareceram apenas uma vez.

A CHD do *corpus* textual resultou na criação de dendrograma com três partições. A primeira partição separou a classe 1 (“Os estereótipos de gênero no contexto social”) das demais. A segunda partição originou a classe 4 (“Intervenções físicas e seus contextos”), enquanto a terceira partição separou as classes 2 (“Experiências pessoais, sociais e a relação com profissionais de saúde”) e 3 (“Sofrimentos relacionados à passabilidade”) (Figura 1).

**Figura 1:** Dendrograma de análise do *corpus* textual das entrevistas. São Paulo, Brasil, 2024.

A classe 1 apresentou 186 STs dos 691 classificados pelo IRAMUTEQ, com uma importância relativa de 26,9% e 149 formas ativas. A classe 2 incluiu 202 STs, com uma importância relativa de 29,2% e 114 formas ativas. A classe 3 possui 111 STs, com uma importância relativa de 16,1% e 109 formas ativas. Por fim, a classe 4 englobou 192 STs, com uma importância relativa de 27,8% e 145 formas ativas.

### Os Estereótipos de Gênero No Contexto Social

Essa classe traz os apontamentos feitos pelos entrevistados com relação ao estereótipo de gênero dentro de um contexto social quanto à performance esperada pela sociedade de homens e mulheres com base em suas identidades de gênero, bem como suas características e comportamentos. Isso é demonstrado pelas palavras que constituem a classe com valor de significância < 0,0001, como “homem” (chi2 75,25), “mulher” (chi2 66,68), “padrão” (chi2 52,99), “sociedade” (chi2 42,53), “pressão” (chi2 26,03) e “aparência” (chi2 16,43).

*Socialmente enxergado como um subproduto, o homem precisa ser lido como uma figura máscula e viril, uma figura bruta, uma figura com uma voz ativa, com características que reforçam esse padrão*. (Trans13)

*A sociedade implica que, para ser homem, precisa não ter peitos e ter barba. Acredito que isso vem da binariedade de gênero, já que, desde quando a gente nasce, nos é imposto algo e um padrão a ser seguido só pelo sexo biológico*. (Trans5)

*A desconstrução do que a gente cresce entendendo sobre o que é ser homem ou mulher é algo contínuo, ainda mais quando falamos de pessoas que são não binárias.* (Trans19)

*Acredito que deva ter uma grande pressão da sociedade com isso, no sentido de, se você se identifica como homem, deveria ter o corpo masculino.* (Trans12)

Experiências pessoais, sociais e a relação com profissionais de saúde

Essa classe é formada por palavras com significância < 0,0001, como “falar” (chi2 54,79), “achar” (chi2 37,56), “geral” (chi2 18,11) e “cabeça” (chi2 18,11).

Os STs que dizem respeito a experiências pessoais demonstram que passar por procedimentos afirmadores de gênero auxiliam no reconhecimento do próprio corpo, proporcionando uma qualidade de vida maior e recursos para lidar com transfobia. Além disso, quanto maior for a sua rede de apoio, a decisão de transicionar é feita com mais propriedade, maior tempo de reflexão e discussões produtivas sobre o assunto.

*O máximo que eu puder transparecer que eu sou um homem, sem ter que abrir a boca para falar isso, melhor. Ao mesmo tempo que eu quero passar por procedimentos para me sentir bem comigo.* (Trans10)

*Eu acho que, quando a gente se sente bem no âmbito pessoal, a gente fica blindado de certa forma a coisas exteriores. Hoje em dia, as coisas me atingem muito menos.* (Trans10)

*Eu acho que o amparo é importante, porque eu sei que não vou passar pelo processo sozinho. Não é só o amparo dentro de casa, mas, tipo, de amigos também.* (Trans3)

Já os STs que focam no âmbito social afirmam que, mesmo quando pessoas trans cedem a procedimentos afirmadores de gênero para diminuírem as violências que sofrem, acaba sendo uma decisão de autopreservação, que lhe asseguram maior estabilidade mental. Não só isso, mas também é necessário acompanhamento psicológico, porque transicionar não é uma garantia de que não haverá mais questões com o próprio corpo ou transfobia.

*Eu não diria que sou uma pessoa muito passável, sofro bastante com essa questão, mas venho tentando entender que o problema não sou eu, e sim as outras pessoas.* (Trans7)

*Eu tinha um pouco de receio antes de começar, não vou negar, não. As pessoas à minha volta colocavam na minha cabeça que eu ia me arrepender, mas eu não tinha dúvida do que eu queria.* (Trans9)

*Eu não vivo em uma caverna, as pessoas assumirem uma coisa de mim me faz mal. Tipo, aqui na minha cabeça.* (Trans3)

Nos STs que se referem aos profissionais de saúde, a maioria dos relatos é positiva quanto à adoção do nome social. Porém, todos afirmaram que tanto profissionais quanto os serviços de saúde propriamente ditos, sejam públicos ou privados, possuem pouca ou nenhuma informação sobre o fluxo de atendimento de uma pessoa trans que busca por procedimentos afirmadores de gênero. Além disso, demonstram que profissionais de saúde possuem pouco ou nenhum interesse em acolher questões de saúde de pessoas trans que não dizem respeito a procedimentos dispostos no processo transexualizador, o que mantém tanto os preconceitos de que pessoas LGBTQIAP+ são grupos de risco de diversas condições de saúde quanto as barreiras no acesso à saúde dessa população.

*Caso de pessoas trans solicitando que utilizassem o nome social para realizar os exames. Mas, no geral, minha pouca experiência foi bem tranquila.* (Trans11)

*Eu acho que faltou sensibilidade e informação sobre os processos. A comunidade médica, às vezes, é um pouco insensível no geral.* (Trans8)

*Tô bem encaminhado, mas foi uma luta para achar um endócrino que atendesse o público trans na minha cidade.* (Trans9)

### Sofrimentos Relacionados à Passabilidade

Essa classe apresenta as palavras “saúde” (chi2 63,14), “hormonal” (chi2 46,44), “transicionar” (chi2 38,06), “decisão” (chi2 36,95), “seguro” (chi2 25,44), “medo” (chi2 23,01) e “estética” (chi2 21,02), com significância < 0,0001. Ela possui STs que dizem sobre como possuir passabilidade faz com que pessoas trans recebam um tratamento mais respeitoso da sociedade e também com que se permitam viver mais momentos livres de medo.

*Antes de começar o tratamento hormonal, eu forçava tanto a voz para ficar mais grossa que tinha dias que eu mal conseguia falar de tanta dor na garganta. Eu não fazia ligações com amigos normalmente, evitava ao máximo áudios, conversava só o extremamente necessário.* (Trans9)

*Não me arrependo de ter tomado essa decisão, porque as interações com a sociedade me afetavam tanto que eu estava entrando em depressão muito forte; não tinha mais vontade de viver.* (Trans7)

*Eu sempre tive e ainda tenho muito medo de andar na rua à noite, mas isso deu uma diminuída desde que eu adquiri certa passabilidade, porque agora o máximo que vai acontecer comigo é roubarem meu celular, não me violentarem de outra forma.* (Trans10)

### Intervenções Físicas E Seus Contextos

As palavras com significância < 0,0001 desta classe são “cirurgia” (chi2 32,65), “expectativa” (chi2 88,24), “Binder” (chi2 25,49), “mastectomia” (chi2 19,03), “hormonização” (chi 75,0) e “dor” (chi2 90,91). Nessa classe, são abordados os cenários das intervenções médicas e cirúrgicas quanto à sua acessibilidade, ao impacto que tem nas vidas de pessoas trans e às expectativas associadas à transição.

*Eu gostaria que a mastectomia e a retirada do útero fossem cirurgias mais acessíveis, porque é muito caro. Sem falar do aspecto social, que essas cirurgias são colocadas como cirurgias plásticas. Pra mim, essas cirurgias são prevenção ao suicídio.* (Trans17)

*Eu fiquei muito contente por realizar a cirurgia, mas tanto a cirurgia quanto o início da hormonização não foram coisas que me deixaram “Meu Deus, que demais! Eu consegui!”, porque eu, particularmente, sinto que eu só estou me ajustando ao meu lugar.* (Trans9)

*Me vestir para ir a faculdade sem o Binder é muito pior que qualquer dor nas costas. Com certeza, a disforia e a questão social influenciam muito nisso tudo.* (Trans16)

## DISCUSSÃO

Os estereótipos de gênero são representações simplificadas de características e papéis atribuídos aos homens e às mulheres com base em suas identidades de gênero, estabelecidos no modo de produção capitalista. Desde então, a sociedade cobra a performance de padrões, como a submissão feminina e sobreposição masculina, e aplica mecanismos específicos de opressão àqueles que diferem da heterocisnormatividade, como o processo de marginalização social compulsória de pessoas trans^(23,24)^. Assim, os estereótipos mantêm a reprodução do modo de produção e da sociabilidade vigente, influenciando diversos aspectos da vida com as regras sociais de feminilidade e masculinidade, que moldam os comportamentos, relacionamentos, expectativas sociais e, até mesmo, escolhas profissionais articuladas com o patriarcado, sexismo, racismo e outros preconceitos estruturais.

Tal fato vai ao encontro do processo de institucionalização da sexualidade nas dimensões da identidade, expressão de gênero e orientação sexual, que mantêm “regras” e “normas” instituídas socialmente, em que qualquer corpo que difere do padrão heterocisnormativo e binário atua como força instituinte, provocando e movimentando essa instituição. Contudo, esse movimento é produtor de sofrimento, dor, desassistência à saúde e morte.

Ainda existem muitas barreiras no acesso aos serviços de saúde por parte da população transmasculina, sendo que o medo de sofrer transfobia foi o fator comum mais citado entre os entrevistados. Seguido dele, estão a desinformação e o despreparo dos profissionais sobre o fluxo de atendimento de uma pessoa trans que busca por procedimento afirmador de gênero, seja em serviços de saúde públicos ou privados. Tal fato corrobora pesquisa desenvolvida com 116 pessoas trans nos Estados Unidos e que aponta para a necessidade de promulgação de políticas de saúde capazes de desconstruírem preconceitos nos serviços de saúde para assegurar a integralidade e humanização da assistência a esse público^(25)^.

Não obstante, pesquisa desenvolvida com 110 enfermeiras(os) brasileiras(os) evidenciou a representação social do preconceito em torno da palavra “travesti”^(26)^, o que explicita a existência de muitos profissionais de saúde que negam atendimento e encaminhamento adequado de pessoas trans por conta de discriminação, enquanto alegam motivos de crença e/ou valores pessoais e ainda falta de qualificação que, consequentemente, intensifica o sofrimento, distanciamento social e barreiras aos serviços de saúde^(26)^. Além disso, não existe preocupação em tornar os ambientes acolhedores para a população transmasculina, demonstrada na ausência de vínculo dessa população com equipes de saúde, que afeta diretamente a efetividade de ações de saúde^(25,26)^.

Tal fato dialoga com a noção de implicação no tocante à instituição sexualidade, ou seja, a relação que os profissionais de saúde estabelecem com essa instituição em seu cotidiano de trabalho. Por vezes, uma implicação atravessada por dogmas e crenças pode ser capaz de gerar interferências que dificultam o processo de análise de implicação, fazendo com que esses profissionais mantenham certa resistência e, consequentemente, sobreimplicados com relação à sexualidade. Dito de outra forma, eles estarão “impermeáveis” a qualquer força instituinte que seja capaz de provocar sua forma instituída de pensar e fazer em saúde no que tange à sexualidade humana.

Outro tópico presente nas entrevistas foi a sensibilização da comunidade médica tanto em questões de demandas gerais quanto de demandas específicas do processo transexualizador. Apesar de pessoas trans possuírem direito a serviços de qualidade, políticas públicas efetivas, profissionais comprometidos com o cuidado integral, com escuta qualificada e compressão sobre suas demandas e necessidades, ainda se deparam com desserviço, sobretudo por problemas estruturais no sistema de saúde e escassez de debates na formação em saúde sobre diversidade sexual e de gênero^(25,27)^.

É uma questão que dialoga com o ativismo de mulheres lésbicas e bissexuais, por conta da invisibilidade e abjeção desses corpos, precariedade e vulnerabilidade para conseguirem reconhecimento de profissionais de saúde^(28)^. Logo, muitos procedimentos médicos/cirúrgicos que deveriam acabar com a disforia e promover passabilidade acabam por denunciar a transição e a transexualidade, seja porque foram feitos de maneira clandestina e com poucos recursos e/ou porque foram realizados por médicos que não se importaram em trazer harmonia para corpos trans e só querem lucrar em cima do desespero pelo procedimento.

Este último apontamento diz muito sobre a sobreimplicação médica daquilo que é estabelecido sobre sexualidade, reforçando a cisgeneridade compulsória na prática profissional, ou seja, sem que exista um processo reflexivo e permanente que revele a fluidez e transitoriedade da sexualidade humana, e as forças instituintes produzidas cotidianamente por meio das diferentes formas de ser e estar no mundo.

As práticas de modificações corporais possuem a finalidade de “conformar” o corpo de uma pessoa trans, no sentido de produzir satisfação pessoal e alcançar materialidade corporal daquilo que ela considera como corpo ideal^(27)^. Seria uma forma de adequação ao instituído no tocante às expressões de gênero binárias e compulsoriamente colocadas como “corretas”. Assim, é imprescindível que procedimentos médico-cirúrgicos dispostos no Processo Transexualizador do SUS considerem cuidadosamente os desejos e expectativas da pessoa trans, avaliando os recursos disponíveis de forma minuciosa e, ao mesmo tempo, reconhecimento por parte do profissional de saúde da autonomia, protagonismo e legitimidade da pessoa em relação ao procedimento. É crucial manter uma visão sensível à biopolítica que permeia esses corpos, porque sofrerão inscrições sociais e simbólicas^(27,29)^. Inclusive, sofrerão não apenas da sociedade cisgênera, mas também dentro da própria comunidade trans, onde existe uma prática de comparação de cirurgias que fomenta competições e julgamentos, ao contrário da celebração e apoio.

O SUS ainda enfrenta muitos desafios para atender à população trans de forma adequada. Na prática, a capacidade de resolução das equipes de saúde sobre as demandas das pessoas trans é muito baixa, porque não há rede de suporte, recursos financeiros destinados a essa questão, preparo técnico das equipes que prestam assistência e nem preparo das próprias organizações no que diz respeito à adoção de fluxos e protocolos cotidianos de atendimento^(30)^. Ainda, o processo transexualizador não foi idealizado para ser eixo de política pública, e o conjunto de normativas, princípios e diretrizes tampouco são é para efetivação de uma^(30)^. Consiste na máxima de que, para que haja um processo de institucionalização em instituições, como a sexualidade, deve-se envolver as pessoas neste processo, visto se tratar de construção efetivamente estabelecida e edificada socialmente.

Vale ressaltar que os serviços de saúde da rede particular sofrem com os mesmos problemas. Porém, nesta via, existe também o fator financeiro e o custo elevado, que inviabilizam o acesso da grande maioria. Como resultado, existe uma considerável atividade clandestina relacionada à venda de hormônios e realização de procedimentos cirúrgicos, da mesma forma que outras tecnologias de saúde perderam sua exclusividade direta com a medicina e começaram a ser capturadas pelo mercado financeiro, muitas vezes por vendedores não profissionais de saúde, possibilitando que muitas pessoas passem por processos de transição de forma desassistida^(30)^.

Este estudo traz à tona as questões relacionadas aos processos de transição vividos por homens trans, que perpassam a busca de procedimentos médicos e atendimento nos serviços de saúde e o enfrentamento social e individuais cotidianos, e que, diante das mazelas sociais em que essas pessoas são alocadas e da perspectiva neoliberal e predatória do desenvolvimento de pesquisas sobre elas e nunca com elas, dificilmente protagonizavam ou participavam de estudos sobre essa temática. Ainda, essa produção avança no olhar para a saúde das pessoas transmasculinas, evidenciando o gargalo ainda existente na saúde pública brasileira para o atendimento às suas necessidades de saúde e que intercepta diversas direções, tais como a necessidade de um olhar atento para a formação de profissionais de saúde e a Educação Permanente em Saúde de profissionais já formados no tocante à saúde de pessoas trans. Apresenta como limitações: o fato de o recrutamento ter ocorrido por meio de formulário virtual, o que possibilitou o seu preenchimento por qualquer pessoa que não, necessariamente, o público-alvo do estudo; as entrevistas terem sido realizadas remotamente (online), o que dificultou sobremaneira a qualidade de suas participações sem que houvesse interrupções de ordem pessoal e/ou da má qualidade da internet; e a diversidade cultural dos participantes da pesquisa que, ao mesmo tempo em que enriqueceu o estudo, trouxe vieses com relação às diferenças existentes no SUS dentro do território nacional.

## CONCLUSÃO

No tocante à percepção de homens trans sobre o processo de transição médica e social, o presente estudo aponta para a dissociação entre influências sociais e influências pessoais no processo de transição vivido por pessoas transmasculinas. A questão da aparência é um dos grandes motivadores, principalmente as alterações físicas proporcionadas pela hormonioterapia e mastectomia. Dessa forma, as expectativas em torno de procedimentos afirmadores de gênero foram de alcançar tanto um estado em que não haja mais disforia com o próprio corpo quanto de harmonia entre a autopercepção e a forma como são percebidos por outras pessoas, minimizando ao máximo as suposições de gênero incongruentes e situações de transfobia.

Isso posto, este estudo identificou que pessoas transmasculinas buscam pela passabilidade adquirida por meio das transições médicas e cirúrgicas, ampliando os recursos mentais para lidar com episódios de violência, principalmente no tocante à autoculpabilização. Somado a isso, a existência de uma rede de apoio ampla e sólida pode favorecer a busca por essas transições, oferecendo suporte emocional e facilitando o diálogo sobre as questões de gênero.

# Translation: Proceso de transición de hombres trans: estereotipos de género, intervenciones y experiencias

**DOI:** 10.1590/1980-220X-REEUSP-2024-0164es

**Authors:** Bruno Torelli de Camargo, Flávio Adriano Borges, José Francisco Sampaio Souza, Natália Sevilha Stofel, Diene Monique Carlos, Márcia Niituma Ogata

**Affiliations:** 1 Universidade Federal de São Carlos, Departamento de Enfermagem, São Carlos, SP, Brasil.; 2 Universidade de São Paulo, Escola de Enfermagem de Ribeirão Preto, Ribeirão Preto, SP, Brasil.

**Keywords:** Minorías Sexuales y de Género, Personas Transgénero, Enfermería, Salud Pública, Análisis Institucional

## Abstract

**Objetivo:**

Analizar la percepción de los hombres trans sobre el proceso de transición.

**Método:**

Estudio cualitativo, desarrollado con personas transmasculinas de febrero a marzo de 2024. Se utilizó un cuestionario y entrevistas semiestructuradas, las cuales fueron grabadas, transcritas y analizadas mediante análisis de contenido. Los hallazgos se discutieron con cinco principios de análisis institucional, como instituido, instituyente, institucionalización, implicación y sobreimplicación.

**Resultados:**

Participaron de las entrevistas 18 personas y el análisis de los datos permitió construir cuatro clases: i) Estereotipos de género en el contexto social; ii) Intervenciones físicas y sus contextos; iii) Experiencias personales, sociales y relaciones con profesionales de la salud; iv) Sufrimientos relacionados con la pasabilidad.

**Conclusión:**

no existe disociación entre influencias sociales y personales en el proceso de transición. Esperan que los procedimientos de afirmación de género reduzcan la disforia y promuevan una armonía entre la autopercepción y la heteropercepción. La pasabilidad adquirida puede fortalecer la capacidad de afrontar la violencia. Además, una red de apoyo amplia y sólida puede favorecer la búsqueda de estas transiciones, facilitando el diálogo sobre cuestiones de género.

## INTRODUCCIÓN

La primera mención de la transexualidad en la literatura la hizo el médico alemán Magnus Hirschfeld en 1910, cuando publicó un estudio de 100 casos de travestis titulado «*Die Travestiten*». En esta obra introdujo el término «transexualismo psíquico». Fue el primer intento de entender la transexualidad desde una perspectiva médica y sociológica, transformándola de una experiencia individual marginada en un problema de salud pública sujeto a intervención médica, incluida la cirugía de reasignación de sexo y los tratamientos hormonales^(1)^.

Según la perspectiva del esencialismo biológico, que ha prevalecido y aún prevalece en gran parte de la sociedad, existe una correspondencia entre sexo y género, en la que el género se considera una consecuencia del sexo (cisgenerismo). Además, se asume que el sexo determina la orientación sexual, considerándose la heterosexualidad como el estándar debido a la reproducción humana. Esta lógica, conocida como cisheteronormatividad, busca controlar los cuerpos bajo la justificación de que es algo natural y biológico, ignorando las influencias culturales y sociales^(2)^.

En cuanto a la transexualidad, también conocida y adoptada en esta producción como la expresión «trans», no existe un estándar fijo de identidad. Se define por la experiencia y la identificación de género de la persona, que puede ser diferente de la asignada al nacer^(3)^.

Durante mucho tiempo, la transexualidad se consideró una patología y se incluyó en la Clasificación Internacional de Enfermedades (CIE). Solo en 2018, con la CIE-11, se eliminó la transexualidad de la lista de trastornos mentales y se categorizó como incongruencia de género. Este cambio estandarizó la identidad de género trans a nivel mundial, aunque se mantuvo en la CIE para garantizar el acceso al tratamiento médico (CIE-10: F-64, CIE-11: HA60), buscando alcanzar el objetivo específico VI de la Política Nacional de Salud Integral para Lesbianas, Gays, Bisexuales, Travestis y Transexuales para garantizar el acceso al proceso transexualizador en la red del Sistema Único de Salud (SUS)^(4)^.

La identidad transmasculina no debe reducirse a la búsqueda de transiciones médicas o quirúrgicas, sino que debe considerarse como una cuestión que desafía los límites entre sexo y género, buscando una comprensión más amplia y fluida de la identidad de género^(5)^, es decir, una identidad que supere la perspectiva de una naturaleza fija e inmutable y considere la existencia de una hibridez capaz de traspasar los dominios artificialmente establecidos de la naturaleza y la cultura^(6)^.

Las estimaciones belgas sugieren que hay 1:12.900 (0,07) mujeres trans en la literatura científica y 1:33.800 (0,02) hombres trans, pero no hay estudios formales que determinen esta estimación en términos concretos^(7)^. En Brasil, aún son escasas y recientes las publicaciones científicas que abordan la atención a la salud de los hombres transexuales^(8,9,10,11)^, y es evidente que la información se produce fuera del círculo científico por medio de boletines, informes, guías y otras producciones autónomas, lo que demuestra que esta población también se sitúa al margen de la producción de conocimiento científico.

Se sabe que las personas transexuales tienden a evitar la búsqueda de atención sanitaria incluso cuando están enfermas^(10,12,13)^ o abandonan el tratamiento propuesto por miedo a la discriminación por parte de los profesionales de salud^(14)^. Además, el acceso a los servicios de salud por parte de la población transexual está impregnado de limitaciones y prejuicios, siendo la exclusión, la impotencia, la omisión y la indiferencia los principales sentimientos expresados por estas personas^(10,14,15)^.

No sólo eso, sino que la gran mayoría de los hombres trans prefieren omitir que son transgénero y vivir como un hombre cis, aprovechando su «pasabilidad», es decir, la posibilidad de ser identificados a través de la expresión de género como un hombre cis y así obtener respeto, protección y derechos básicos(5) , así como autoadministrarse esteroides a base de testosterona sin el debido control médico o sanitario^(16)^. En este contexto, surge la pregunta: ¿cómo perciben los hombres trans el proceso de transición médica y social? Este estudio es crucial para ampliar nuestra comprensión del tema, sacando a la luz los puntos de vista de las personas que viven a diario los problemas del proceso de transición de género. Por lo tanto, este estudio pretende analizar las percepciones de los hombres trans sobre el proceso de transición.

## MÉTODO

### Diseño del Estudio

Se trata de un estudio descriptivo y exploratorio con enfoque cualitativo, que siguió en su desarrollo las recomendaciones establecidas por los *COnsolidated criteria for REporting Qualitative* research y buscó significados que articularan la teoría, la práctica y la investigación posterior, además de proporcionar una observación de múltiples significados, motivos, aspiraciones, creencias, valores y actitudes, posibilitando la comprensión de un espacio más profundo de relaciones, procesos y fenómenos^(17)^.

### Localización, Población y Criterios de Selección y Definición de la Muestra

La investigación se realizó en Brasil, con hombres trans y/o personas trans no binarias alineadas con el género masculino. Los criterios de inclusión de participantes en el estudio fueron ser hombre trans y/o persona trans no binaria alineada con el género masculino, residir en Brasil y haberse sometido o no a procedimientos de transición. Los criterios de exclusión fueron no rellenar el formulario de la encuesta en su totalidad, dejarlo incompleto y no responder al correo electrónico solicitando una entrevista después del quinto intento.

Se publicitó a través de las redes sociales de los investigadores (*Facebook^®^*, *Instagram^®^* y *WhatsApp^®^*), seguida de la técnica de *snowballing* (bola de nieve*)*, que utiliza cadenas de referencia para su ejecución, es decir, después de que un participante haya completado la encuesta, remite a otros participantes a la misma, publicitando el formulario a través del mismo *enlace* puesto a su disposición^(18)^.

Esta técnica puede utilizarse en estudios con grupos de difícil acceso o cuando la investigación se refiere a asuntos privados. Esta investigación cumple ambos criterios para utilizar esta técnica, que aprovecha la red de contactos de los sujetos para proporcionar al investigador un mayor conjunto de contactos potenciales^(18)^. Además, se entiende que *el snowballing* es una técnica capaz de garantizar la no exposición de los individuos, preservar la capacidad de decisión de los participantes, reforzar el respeto a la diversidad y la participación de grupos vulnerables y discriminados, como es el caso de los hombres trans.

La muestra se finalizó según el criterio de saturación de datos, es decir, hasta que no hubiera nuevos temas o informaciones - procedentes de las entrevistas - en el marco del análisis del objeto de estudio. La saturación de datos se alcanzó cuando se identificó una combinación de los límites empíricos de los datos, su relación con el marco teórico de la investigación y su articulación con la sensibilidad de los investigadores que lo desarrollaron^(19)^. De esta forma, la saturación de los datos se llevó a cabo de acuerdo con el rigor metodológico y científico, que implica el análisis continuo de los datos (desde el inicio del proceso de recolección de datos), con este análisis preliminar buscando exactamente el momento en que aparecerá poca novedad sustancial, con el fin de añadir al objetivo del estudio, respondiendo a su pregunta orientadora^(19)^. *Recogida de datos*

La recogida de datos se realizó entre febrero y marzo de 2024, mediante un cuestionario autoadministrado en formato electrónico, con datos sociodemográficos, preguntas específicas del objeto de estudio, publicado en redes sociales, y entrevistas semiestructuradas realizadas a distancia (*online*).

Una vez cumplimentado el formulario en su totalidad, se contactó con los participantes para concertar una entrevista semiestructurada. El orden de reclutamiento para la entrevista se basó en las regiones del país y obedeció al orden cronológico en que se rellenó el formulario, es decir, el reclutamiento comenzó con el primer participante del Sudeste, que rellenó el formulario, luego el Nordeste, y así sucesivamente, hasta alcanzar la saturación de datos. Las preguntas utilizadas en las entrevistas fueron previamente presentadas al grupo de investigación (formado por personas cis y trans y con una amplia diversidad étnico-racial) con el fin de mejorarlas, para que pudieran ser utilizadas en esta investigación: En su opinión, ¿qué lleva a las personas transmasculinas a someterse a procedimientos médicos quirúrgicos? Si quieres someterte a un procedimiento de afirmación de género, ¿qué esperas de él? En caso negativo, ¿por qué no? ¿Cómo suelen tratarle los profesionales de salud? Cabe señalar que éstas eran preguntas clave, que se desdoblaron en otras preguntas en la dirección del objeto del estudio.

### Análisis y Tratamiento de los Datos

Las entrevistas duraron una media de 30 minutos y fueron grabadas y transcritas en su totalidad, procediéndose al análisis de contenido^(19)^.

El análisis de contenido permite construir categorías a partir de agrupaciones semánticas de palabras en frases. Exige sensibilidad y flexibilidad por parte del codificador para aprehender los núcleos temáticos capaces de componer el sentido de la comunicación deseada^(20)^. Se compone de las siguientes etapas: 1) preanálisis, que incluye la composición del *corpus* textual, la lectura flotante y la definición de hipótesis provisionales sobre el contenido leído; 2) exploración del material, en la que se codificarán los datos a partir de las unidades de registro; y 3) tratamiento de los resultados e interpretación, que consiste en la clasificación de los elementos a partir de sus semejanzas y por diferenciación, con posterior agrupación en función de sus características comunes^(20)^.

Cabe mencionar que para la etapa 2 y parte de la etapa 3, se utilizó el *programa informático Interface de R pour les Analyses Multidimensionnelles de Textes et de Questionnaires* (IRAMUTEQ^®)^, que utiliza el Método de Reinert para construir la Clasificación Jerárquica Descendente (CJD). El *software* realiza un análisis basado en la agrupación de palabras con similitud semántica presentes en el *corpus* textual. Este *corpus* se divide en segmentos textuales (ST), que consisten en pequeños fragmentos textuales que conservan una relación semántica entre sí^(21)^. El método organiza las formas léxicas en clases, asignando a cada una de ellas una importancia relativa. Las ocurrencias de cada una de las clases en la CJD se consideraron a partir de valores estadísticamente significativos (p < 0,05).

Los resultados se compararon con algunos principios del análisis institucional, como la institución y sus tres momentos – instituido, instituyente e institucionalización -, la implicación y la sobreimplicación. La institución corresponde a las normas y reglas creadas y establecidas socialmente, siendo lo instituido lo claro e identificable en esa institución; lo instituyente es lo que desplaza, mueve y provoca lo instituido; y la institucionalización corresponde al proceso dialéctico entre lo instituido y lo instituyente^(22)^. La implicación, por su parte, consiste en la relación que las personas establecen con las instituciones, lo que puede ocurrir de diferentes maneras y desde diferentes perspectivas, y lo que dificulta el proceso de análisis de esa implicación corresponde a la sobreimplicación^(22)^.

Siempre en diálogo con el análisis institucional, partimos del supuesto de que el investigador nunca es neutral en la elección de su objeto de estudio, y siempre está implicado con él, ya sea desde una perspectiva ideológica, libidinal y/o organizacional. Así, los autores aquí presentes asumen sus implicaciones con el objeto de este estudio, por ser profesionales de la salud e investigadores en el área de las minorías sexuales y de género desde diferentes perspectivas (atención a la salud, prejuicios, violencia, etc.) y también porque la mayoría de los autores pertenecen a la comunidad LGBT+, es decir, lesbianas, gays, bisexuales, transexuales y otras identidades de género u orientaciones sexuales que difieren del estándar hetero-cis-normativo.

### Aspectos Éticos

La investigación fue aprobada por el Comité de Ética para la Investigación con Seres Humanos de la Universidad Federal de São Carlos, bajo el Dictamen n° 5.985.157 y el Certificado de Sometimiento a Evaluación Ética n° 63925222.2.0000.5504. Cabe destacar que, antes de rellenar el cuestionario, el participante tuvo acceso al Formulario de Consentimiento Libre e Informado, expresando su conformidad para participar en la investigación. Para garantizar el anonimato de los participantes en la investigación, las palabras pronunciadas por los participantes se identificaron con la expresión «Trans», seguida del número correspondiente.

## RESULTADOS

Un total de 88 hombres trans y/o personas trans no binarias alineadas con el género masculino completaron el cuestionario autoadministrado. De ellos, tres fueron excluidos por no cumplir los criterios de inclusión del estudio, y 67 no fueron reclutados para la entrevista debido a la saturación de datos, que se produjo en la 18^a^ entrevista. Por lo tanto, 18 hombres trans y/o personas trans no binarias alineadas con el género masculino, que se encontraban antes, después o durante la transición médica/social o que optaron por no someterse a ninguna de las transiciones, participaron en la fase de entrevista semiestructurada. Cabe destacar que ningún participante, al ser reclutado para la entrevista, se negó y/o fue excluido de la investigación (Tabla 1).

**Tabla 1:** Caracterización sociodemográfica de los hombres trans y/o personas trans no binarias alineadas al género masculino que participaron del estudio – São Carlos, SP, Brasil, 2024.

Orientación sexual	Ciudad/Estado	Ingresos (salario mínimo)	Color/etnia	Nivel de estudios	Edad (años)
Pansexual	Río de Janeiro/RJ	1/2 a 1	Indígena	Estudios superiores terminados	21 a 25
Pansexual	Campinas/SP	1 a 2	Pardo	Educación superior incompleta	21 a 25
Pansexual	São Paulo/SP	1 a 2	Blanco	Estudios superiores terminados	21 a 25
Bisexual	Betim/MG	1/2 a 1	Negro	Educación superior incompleta	21 a 25
Pansexual	Feira de Santana/BA	2 a 3	Blanco	Estudios superiores terminados	21 a 25
Bisexual	Fortaleza/CE	1/2 a 1	Amarillo	Educación superior incompleta	21 a 25
Asexual	Salvador/BA	Sin ingresos	Pardo	Bachillerato completo	16 a 20
Bisexual	Sobral/CE	2 a 3	Blanco	Bachillerato completo	16 a 20
Bisexual	Curitiba/PR	1 a 2	Blanco	Postgrado completo	26 a 30
Asexual	Passo Fundo/RS	Sin ingresos	Blanco	Educación superior incompleta	21 a 25
Gay	Bocaina do Sul/SC	1/2 a 1	Blanco	Bachillerato incompleto	16 a 20
Pansexual	Florianópolis/SC	Sin ingresos	Blanco	Educación superior incompleta	21 a 25
Gay	Manaos/AM	1 a 2	Blanco	Educación superior incompleta	21 a 25
Gay	Belém/PA	1 a 2	Blanco	Estudios superiores terminados	16 a 20
Pansexual	Boa Vista/RR	Más del 3	Blanco	Educación superior incompleta	16 a 20
Heterosexual	Cuiabá/MT	1/2 a 1	Negro	Educación superior incompleta	21 a 25
Heterosexual	Rondonópolis/MT	2 a 3	Blanco	Estudios superiores terminados	16 a 20
Bisexual	Três Lagoas/MS	Más de 3	Blanco	Educación superior incompleta	16 a 20

Fuente: datos de la investigación.

Durante la parametrización del *corpus* textual, se analizaron 18 textos, correspondientes a las 18 entrevistas realizadas a hombres trans, y se dividieron en 854 ST. De ellos, 691 fueron clasificados con un 80,91%, lo que indica que los entrevistados mantuvieron la atención en el tema principal y no mostraron variaciones significativas de vocabulario. Además, se identificaron un total de 28.478 formas léxicas, de las cuales sólo 3.578 eran distintas y 1.833 de ellas aparecían una sola vez. El CJD del *corpus de* textos dio lugar a la creación de un dendrograma con tres particiones. La primera partición separó la clase 1 («Estereotipos de género en el contexto social») de las demás. La segunda partición dio lugar a la clase 4 («Intervenciones físicas y sus contextos»), mientras que la tercera separó las clases 2 («Experiencias personales, sociales y la relación con los profesionales de salud») y 3 («Sufrimientos relacionados con la pasabilidad») (Figura 1).

**Figura 1:** Dendrograma de análisis del *corpus* textual *de* las entrevistas. São Paulo, Brasil, 2024.

La clase 1 presentaba 186 ST de las 691 clasificadas por el IRAMUTEQ, con una importancia relativa del 26,9% y 149 formas activas. La clase 2 incluía 202 ST, con una importancia relativa del 29,2% y 114 formas activas. La clase 3 tiene 111 ST, con una importancia relativa del 16,1% y 109 formas activas. Por último, la clase 4 incluye 192 ST, con una importancia relativa del 27,8% y 145 formas activas.

### Estereotipos de Género en el Contexto Social

Esta clase contiene los comentarios de los entrevistados sobre los estereotipos de género en el contexto social, en relación con la actuación que la sociedad espera de hombres y mujeres en función de sus identidades de género, así como de sus características y comportamientos. Así lo demuestran las palabras que componen la clase con un valor de significación < 0,0001, como «hombre» (chi2 75,25), «mujer» (chi2 66,68), «norma» (chi2 52,99), «sociedad» (chi2 42,53), «presión» (chi2 26,03) y «apariencia» (chi2 16,43).

Visto socialmente como un subproducto, los hombres necesitan ser leídos como una figura viril y varonil, una figura ruda, una figura con voz activa, con características que refuercen este estándar. (Trans13)

*La sociedad da a entender que para ser hombre hay que carecer de pechos y tener barba. Creo que esto proviene del binario de género, ya que desde el momento en que nacemos, se nos impone algo y un patrón a seguir basado únicamente en nuestro sexo biológico.* (Trans5)

*Deconstruir lo que crecemos entendiendo sobre lo que significa ser hombre o mujer es un proceso continuo, especialmente cuando hablamos de personas no binarias.* (Trans19)

*Creo que debe haber mucha presión por parte de la sociedad en este sentido, en el sentido de que si te identificas como hombre, debes tener un cuerpo masculino.* (Trans12)

### Experiencias Personales y Sociales y Relación con los Profesionales de Salud

 Esta clase está formada por palabras con significación < 0,0001, como «hablar» (chi2 54,79), «encontrar» (chi2 37,56), «general» (chi2 18,11) y «cabeza» (chi2 18,11).

Los ST que se refieren a experiencias personales muestran que someterse a procedimientos de afirmación de género ayuda a reconocer el propio cuerpo, lo que proporciona una mejor calidad de vida y recursos para hacer frente a la transfobia. Además, cuanto mayor es su red de apoyo, la decisión de hacer la transición se toma con más propiedad, más tiempo para la reflexión y debates productivos sobre el tema.

*Cuanto más pueda demostrar que soy un hombre, sin tener que abrir la boca para decirlo, mejor. Al mismo tiempo, quiero seguir los procedimientos para sentirme bien conmigo mismo*. (Trans10)

*Creo que cuando te sientes bien contigo mismo personalmente, te blindas un poco contra las cosas externas. Hoy en día, las cosas me afectan mucho menos*. (Trans10)

*Creo que el apoyo es importante, porque sé que no voy a pasar por el proceso sola. No es sólo el apoyo en casa, sino también el de los amigos.* (Trans3)

Los ST que se centran en el ámbito social afirman que incluso cuando las personas trans ceden a los procedimientos de afirmación de género para reducir la violencia que sufren, acaba siendo una decisión de autopreservación que garantiza una mayor estabilidad mental. No sólo eso, sino que el asesoramiento psicológico también es necesario, porque la transición no es una garantía de que no habrá más problemas con el propio cuerpo o transfobia.

*No diría que soy una persona muy trans, sufro mucho por este tema, pero he intentado entender que el problema no soy yo, sino otras personas.* (Trans7)

*Tenía un poco de miedo antes de empezar, no lo voy a negar. La gente a mi alrededor me decía que me arrepentiría, pero yo no tenía ninguna duda sobre lo que quería*. (Trans9)

*No vivo en una cueva, que la gente suponga algo de mí me hace daño. Como aquí en mi cabeza.* (Trans3)

En los ST que se refieren a profesionales de salud, la mayoría de los informes son positivos sobre la adopción de un nombre social. Sin embargo, todos ellos afirman que tanto los profesionales como los propios servicios de salud, ya sean públicos o privados, tienen poca o ninguna información sobre el flujo de atención a una persona trans que solicita procedimientos de afirmación de género. Además, demuestran que los profesionales de la salud tienen poco o ningún interés en tratar los problemas de salud de las personas trans que no tienen que ver con los procedimientos establecidos en el proceso de transexualización, lo que mantiene tanto el prejuicio de que las personas LGBTQIAP+ corren el riesgo de sufrir diversas afecciones de salud como las barreras de acceso a la atención sanitaria para esta población.

*Se han dado casos de personas trans que piden usar su nombre social para hacerse pruebas. Pero en general, mi pequeña experiencia fue bastante tranquila»* (Trans11)

*Creo que faltó sensibilidad e información sobre los procesos. La comunidad médica es a veces un poco insensible en general.»* (Trans8)

*Estoy bien encaminada, pero me costó encontrar un endocrino que atendiera a personas trans en mi ciudad.* (Trans9)

### Sufrimientos Relacionados con la Pasabilidad

 Esta clase incluye las palabras «salud» (chi2 63,14), «hormonal» (chi2 46,44), «transición» (chi2 38,06), «decisión» (chi2 36,95), «seguro» (chi2 25,44), «miedo» (chi2 23,01) y «estética» (chi2 21,02), con significación < 0,0001. Hay algunos STs que dicen cómo tener pasabilidad hace que las personas trans reciban un trato más respetuoso por parte de la sociedad y también les permite vivir más momentos libres de miedo.

*Antes de empezar el tratamiento hormonal, forzaba tanto la voz para hacerla más gruesa que había días en los que apenas podía hablar por el dolor de garganta. No hacía llamadas telefónicas con amigos normalmente, evitaba los audios en la medida de lo posible, sólo hablaba cuando era extremadamente necesario»* (Trans9).

*No me arrepiento de haber tomado esta decisión, porque las interacciones con la sociedad me afectaban tanto que estaba cayendo en una depresión muy grave; ya no tenía ganas de vivir.* (Trans7)

*Siempre he tenido y tengo mucho miedo de caminar por la calle de noche, pero esto ha disminuido desde que adquirí cierta pasabilidad, porque ahora lo más que me va a pasar es que me roben el móvil, no que me violen de ninguna otra manera.* (Trans10)

### Intervenciones Físicas y sus Contextos

 Las palabras con significación < 0,0001 en esta clase son «cirugía» (chi2 32,65), «expectativa» (chi2 88,24), «carpeta» (chi2 25,49), «mastectomía» (chi2 19,03), «hormonización» (chi 75,0) y «dolor» (chi2 90,91). En esta clase se abordan los escenarios de las intervenciones médicas y quirúrgicas en cuanto a su accesibilidad, el impacto que tienen en la vida de las personas trans y las expectativas asociadas a la transición.

*Me gustaría que la mastectomía y la extirpación del útero fueran cirugías más accesibles, porque son muy caras. Por no hablar del aspecto social de que estas cirugías se etiqueten como cirugía plástica. Para mí, estas cirugías son la prevención del suicidio*. (Trans17)

*Me alegré mucho de operarme, pero tanto la operación como el inicio de la terapia hormonal no fueron cosas que me hicieran decir «¡Dios mío, es increíble! Lo logré!», porque particularmente siento que me estoy adaptando a mi lugar.* (Trans9)

*Vestirse para ir a la universidad sin Binder es mucho peor que cualquier dolor de espalda. Sin duda, la disforia y las cuestiones sociales influyen mucho en todo esto.* (Trans16)

## DISCUSIÓN

Los estereotipos de género son representaciones simplificadas de características y roles atribuidos a hombres y mujeres en función de sus identidades de género, establecidas en el modo de producción capitalista. Desde entonces, la sociedad ha exigido el cumplimiento de normas, como la sumisión femenina y la superposición masculina, y ha aplicado mecanismos específicos de opresión a quienes difieren de la heterocisnormatividad, como el proceso de marginación social obligatoria de las personas trans^(23,24)^. De este modo, los estereotipos mantienen la reproducción del actual modo de producción y sociabilidad, influyendo en diversos aspectos de la vida con las reglas sociales de la feminidad y la masculinidad, que moldean el comportamiento, las relaciones, las expectativas sociales e incluso las opciones profesionales en conjunción con el patriarcado, el sexismo, el racismo y otros prejuicios estructurales.

Esto va de la mano con el proceso de institucionalización de la sexualidad en las dimensiones de identidad, expresión de género y orientación sexual, que mantienen «reglas» y «normas» socialmente instituidas, en las que cualquier cuerpo que difiera del estándar heterocisnormativo y binario actúa como fuerza instituyente, provocando y moviendo esta institución. Sin embargo, este movimiento produce sufrimiento, dolor, falta de atención sanitaria y muerte.

Todavía existen muchas barreras de acceso a los servicios de salud para la población transmasculina, siendo el miedo a sufrir transfobia el factor más citado entre los entrevistados. Le siguen la desinformación y la falta de preparación por parte de los profesionales sobre el flujo de atención a una persona trans que busca un procedimiento de afirmación de género, ya sea en servicios de salud públicos o privados. Esto corrobora la investigación realizada con 116 personas trans en los Estados Unidos, que apunta a la necesidad de promulgar políticas de salud capaces de deconstruir los prejuicios en los servicios de salud para garantizar una atención integral y humanizada a este público^(25)^.

Sin embargo, una encuesta realizada a 110 enfermeras brasileñas mostró la representación social del prejuicio en torno a la palabra «travesti»^(26)^, lo que deja claro que muchos profesionales de la salud niegan la atención y la derivación adecuada a las personas trans por discriminación, alegando razones de creencias y/o valores personales y falta de cualificación, lo que, en consecuencia, intensifica el sufrimiento, el distanciamiento social y las barreras a los servicios de salud^(26)^. Además, no hay preocupación por hacer ambientes acogedores para la población transmasculina, demostrada por la falta de vínculo entre esta población y los equipos de salud, lo que afecta directamente la eficacia de las acciones de salud^(25,26)^.

Esto dialoga con la noción de implicación en relación a la institución de la sexualidad, es decir, la relación que los profesionales de salud establecen con esta institución en su trabajo cotidiano. En ocasiones, una implicación atravesada por dogmas y creencias puede generar interferencias que dificulten el proceso de análisis de la implicación, haciendo que estos profesionales mantengan cierta resistencia y, en consecuencia, se sobreimpliquen en relación con la sexualidad. En otras palabras, serán «impermeables» a cualquier fuerza instituyente que pueda provocar su forma establecida de pensar y hacer en salud cuando se trata de la sexualidad humana.

Otro tema que surgió en las entrevistas fue la conciencia de la comunidad médica tanto de las demandas generales como de las demandas específicas del proceso de transexualización. A pesar de que las personas trans tienen derecho a servicios de calidad, políticas públicas eficaces, profesionales comprometidos con la atención integral, con escucha cualificada y compresión de sus demandas y necesidades, todavía se enfrentan a un mal servicio, principalmente debido a problemas estructurales del sistema de salud y a la falta de debates en la formación sanitaria sobre la diversidad sexual y de género^(25,27)^.

Esta es una cuestión que dialoga con el activismo de las mujeres lesbianas y bisexuales, debido a la invisibilidad y abyección de estos cuerpos, su precariedad y vulnerabilidad para obtener el reconocimiento de los profesionales de la salud^(28)^. Por lo tanto, muchos procedimientos médico-quirúrgicos que deberían acabar con la disforia y promover la pasibilidad acaban denunciando la transición y la transexualidad, ya sea porque se hicieron de forma clandestina y con pocos recursos y/o porque fueron realizados por médicos a los que no les importaba traer armonía a los cuerpos trans y sólo querían lucrar con la desesperación del procedimiento.

Este último punto dice mucho sobre la sobreimplicación médica de lo establecido sobre la sexualidad, reforzando el cisgenerismo obligatorio en la práctica profesional, o sea, sin que haya un proceso reflexivo y permanente que revele la fluidez y transitoriedad de la sexualidad humana, y las fuerzas instituyentes producidas cotidianamente a través de las diferentes formas de estar en el mundo.

Las prácticas de modificación corporal tienen como objetivo «conformar» el cuerpo de una persona trans para producir satisfacción personal y alcanzar la materialidad corporal de lo que consideran el cuerpo ideal^(27)^. Sería una forma de adaptarse a lo instituido en términos de expresiones binarias de género que se colocan obligatoriamente como «correctas». Por lo tanto, es fundamental que los procedimientos médico-quirúrgicos previstos en el Proceso de Transexualización del SUS consideren cuidadosamente los deseos y expectativas de la persona trans, evaluando detalladamente los recursos disponibles y, al mismo tiempo, reconociendo la autonomía, el protagonismo y la legitimidad del profesional de salud en relación al procedimiento. Es crucial mantener una mirada sensible a la biopolítica que permea esos cuerpos, pues sufrirán inscripciones sociales y simbólicas^(27,29)^. Sufrirán no sólo de la sociedad cisgénero, sino también dentro de la propia comunidad trans, donde existe una práctica de comparación de cirugías que fomenta la competencia y el juicio, en contraposición a la celebración y el apoyo.

El SUS aún se enfrenta a muchos desafíos para proporcionar una atención adecuada a la población trans. En la práctica, la capacidad de los equipos de salud para resolver las demandas de las personas trans es muy baja, porque no existe una red de apoyo, no hay recursos financieros destinados a esta cuestión, no hay capacitación técnica para los equipos que prestan atención y no hay capacitación para las propias organizaciones en cuanto a la adopción de flujos y protocolos de atención diaria^(30)^. Además, el proceso de transexualización no fue concebido para ser el eje de una política pública, ni el conjunto de reglamentos, principios y directrices destinados a hacerla efectiva^(30)^. Consiste en la máxima de que para que haya un proceso de institucionalización en las instituciones, como la sexualidad, las personas deben estar involucradas en este proceso, ya que es una construcción que efectivamente se establece y se construye socialmente.

Vale la pena subrayar que los servicios de salud privados sufren los mismos problemas. Sin embargo, también existe el factor financiero y el elevado coste, que hacen inviable el acceso para la gran mayoría. Como resultado, existe una considerable actividad clandestina relacionada con la venta de hormonas y la realización de procedimientos quirúrgicos, del mismo modo que otras tecnologías sanitarias han perdido su exclusividad directa con la medicina y han empezado a ser captadas por el mercado financiero, a menudo por vendedores no profesionales de la salud, lo que permite a muchas personas pasar por procesos de transición de forma desasistida^(30)^.

Este estudio saca a la luz las cuestiones relacionadas con los procesos de transición vividos por los hombres trans, que implican la búsqueda de procedimientos médicos y atención en los servicios de salud y la confrontación social e individual cotidiana, y que, dados los males sociales en los que se encuentran estas personas y la perspectiva neoliberal y depredadora de desarrollar investigaciones sobre ellas y nunca con ellas, apenas han ocupado un lugar central o han participado en los estudios sobre este tema. Además, esta producción profundiza en la salud de las personas transmasculinas, destacando el cuello de botella que aún existe en la sanidad pública brasileña a la hora de atender sus necesidades sanitarias, y que atraviesa varias direcciones, como la necesidad de profundizar en la formación de los profesionales de salud y en la Educación Permanente de los profesionales ya formados en la salud de las personas trans. Entre sus limitaciones se destacan: el hecho de que el reclutamiento se realizó por medio de un formulario virtual, lo que significaba que podía ser rellenado por cualquier persona que no fuera necesariamente el público objetivo del estudio; el hecho de que las entrevistas se realizaron a distancia (online), lo que dificultó enormemente garantizar la calidad de su participación sin interrupciones personales y/o la mala calidad de internet; y la diversidad cultural de los participantes de la investigación que, si bien enriqueció el estudio, trajo consigo sesgos en relación a las diferencias que existen en el SUS dentro del territorio nacional.

## CONCLUSIÓN

Con relación a la percepción de los hombres trans sobre el proceso de transición médica y social, este estudio señala la disociación entre las influencias sociales y las influencias personales en el proceso de transición vivido por las personas transmasculinas. La cuestión de la apariencia es un motivador importante, especialmente los cambios físicos provocados por la terapia hormonal y la mastectomía. Así, las expectativas en torno a los procedimientos de afirmación de género eran alcanzar tanto un estado en el que ya no hubiera disforia con el propio cuerpo como una armonía entre la autopercepción y la forma en que son percibidos por otras personas, minimizando en la medida de lo posible las suposiciones de género incongruentes y las situaciones transfóbicas. Dicho esto, este estudio identificó que las personas transmasculinas buscan la pasabilidad adquirida a través de transiciones médicas y quirúrgicas, ampliando sus recursos mentales para lidiar con episodios de violencia, especialmente en términos de autoculpabilización. Además, la existencia de una red de apoyo amplia y sólida puede favorecer la búsqueda de esas transiciones, ofreciendo apoyo emocional y facilitando el diálogo sobre cuestiones de género.
